# Accelerated Life Testing of a Palladium-Doped Tin Oxide Electrode for Zn Electrowinning

**DOI:** 10.3390/ma13081969

**Published:** 2020-04-23

**Authors:** Jung Eun Park, Ho Kim, Eun Sil Lee

**Affiliations:** Center for Plant Engineering, Institute for Advanced Engineering, Yongin-si 17180, Korea; jepark0123@gmail.com (J.E.P.); hokim0505@gmail.com (H.K.)

**Keywords:** electrowinning, palladium-based electrodes, mixed metal oxide, accelerated life testing (ALT)

## Abstract

Electrowinning is a technique that can be used to obtain high-purity elements through electrolysis. The degradation of accelerated life testing for Pd-based electrodes is discussed in this study. The lifetime of the electrodes was examined by multiplying the acceleration rate with the current to measure the voltage of the electrodes. The acceleration rate was set to 10, 20, and 30 times. Four components were deposited on the TiO_2_ plate. The ratio of Ir to Sn was fixed at 1:1, while Ta was deposited at 10 wt.%. Pd was deposited at 2, 4, 8 and 10 wt.% to create Pd-Ir/Sn-Ta. The initial voltage decreased as the Pd deposition amount increased irrespective of the acceleration rate. The lower the acceleration rate, the lower the voltage. An increase in the Pd content caused the initial voltage to be low. The multiple of the acceleration rate slightly increased for all cases of life testing for one year. When the test was conducted by increasing the current density by 20 times, the increase in voltage was proportional to the Pd deposition amount. However, for the 30 times acceleration rate, the lifetime of the electrodes was shortened as the Pd content increased. It can be inferred that the content of Pd and the ratio of Ir to Sn can influence the lifetime of the electrodes. According to these results, if the multiple of the acceleration rate is too extreme, the lifetime of the electrodes cannot be evaluated because they are damaged in an extreme situation.

## 1. Introduction

Rare metallic elements are both difficult to extract and are not readily available around the world. They are at risk of being in short supply due to the concentration of deposits and the production being restricted to certain countries, or due to the import of bulk quantities by specific countries. These rare metals are in demand by industries, and their demand will increase significantly in the future as green and high-tech industries advance [1,2]. However, 80% of the rare metal reserves are only located in certain countries, and the price of rare metals is rising due to the instability in supply caused by the strengthening of the regulations for protecting the natural resources of these countries [3,4]. Due to the limited amount of metal deposits, many studies are being continuously conducted on recycling, reusing, and reducing the use of rare metals, which are valuable industrial resources [5,6,7,8].

Electrowinning is often used in metal refining by collecting metals from industrial wastewater that contains metal substances, resulting in environmental and economic benefits. The principle of electrowinning is to obtain high purity metals by electrolysis [9,10,11]. When metals containing impurities are placed on the anodic side while another metal is placed on the cathodic side, and a current is passed through, high purity metals (Zn, Cu) are extracted on the cathodic side. Many efforts have been made over the past two decades to improve the efficiency of electrowinning [12,13,14]. Various types of technologies have been introduced; however, electrowinning lacks in performance and economic feasibility. In particular, many studies have been conducted on metal compositions; however, most of these investigations focus on simple compositions or characteristics of the electrodes [15,16].

Electrowinning is typically conducted within 48 h when considering the current efficiency and zinc recovery efficiency, in which the electrodes are reused several times. These electrodes, with a high rate of reusability, are provided with a low power consumption in the electrowinning process. When a purified solution undergoes direct electrowinning, the purity of zinc ingot, which is the final product, may be reduced due to various metal substances (excluding zinc) in the purified solution. Therefore, the process of extracting zinc alone from a purified solution and then proceeding with electrowinning is gaining attention. Zinc is then dissolved in sulfuric acid. If the electrodes are exposed to highly concentrated sulfuric acid for an extended period of time, substances coated on the electrodes detach, and zinc recovery is hindered. In addition, the power consumption is increased due to the increased voltage. Thus, the lifetime of electrodes is very important in the electrowinning process [17,18,19].

For electro refining, an excellent electrode must be able to work efficiently for several years. However, testing the electrode stability under normal conditions is time consuming. The lifetime of the electrode is one of the most important factors for the stability of the electrode [20]. Generally, the researcher compared the service life with different compositions of electrodes in 3M H_2_SO_4_ solution [21,22] and suggested the relationship between the service life and deactivation mechanism [23]. The equation of service life (SL = 1/*n^i^*, *I* = 1.7, *n* = current density) is not matched by the results of the accelerated life testing pattern. In recent literature, Bi et al. compared the accelerated lifetime for Ti-based electrodes studied via other experimental results [24]. However, it is hard to compare the accelerated lifetime because of containing various factors. The conditions are mostly low current density (100–1000 mA/cm^2^) and low concentration (0.25–1 M H_2_SO_4_). Since the lifetime of the electrode is considerable, accelerated life testing is required to reduce the testing time. Therefore, accelerated life testing (ALT) methods are likely being adopted in an attempt to build on the collaborative efforts between industry and academia.

Accelerated testing methods are becoming standard practice in many research laboratories. This is especially true for research groups investigating the electrode lifetime. However, as most studies on electrodes are focused on the performance, with no standard methods or guidelines for ALT, diverse test methods are being adopted based on the increase in the operating voltage.

This work is aimed towards comparatively identifying the acceleration life testing for evaluating the performance and reliability of electrodes in an aqueous electrochemical test. In this study, the test methods and equipment were prepared based on the ALT methods suggested by Park [25]. The variables, excluding the electrodes, have been minimized for the test method and equipment. In addition, the complete relationship diagram of electrodes has been compared.

## 2. Experimental Methods

### 2.1. Electrodes

The palladium-based electrodes were manufactured using a dip-coating method. After dip-coating, the specimen was dried for 24 h at 105 °C. The manufactured Pd-based electrodes were provided by West Co. (Changwon-si, Korea). The substrate was a titanium plate, which contained an Ir and Sn manufacturing ratio of 1:1, and a Ta coating weight of 10%. In addition, Pd was added at 2, 4, 8 and 10 wt.%. The Pd-IrSnTa/TiO_2_ electrode contained PdO*_x_*, IrO*_x_*, SnO*_x_* and TaO*_x_* on a TiO_2_ substrate [26,27]. The surface texture and morphology of the Pd-based electrode was characterized using a Field Emission Scanning Electron Microscope (FE-SEM), equipped with an energy dispersive spectrometer (EDS), which was acquired using a Regulus 8230 (HITACHI instrument, Tokyo, Japan) that worked with an acceleration voltage of 10 kV. The surface roughness was analyzed with the scanning probe image processor SmartScan^TM^ (Park systems, California, CA, USA) and XEI software (4.3.0. Build5) by NX 10 (Park systems). The surface roughness parameters such as *R*a (arithmetical mean deviation) and *R*q (root mean square deviation) were estimated [28].

### 2.2. Accelerated Life Testing (ALT)

**Figure 1:**
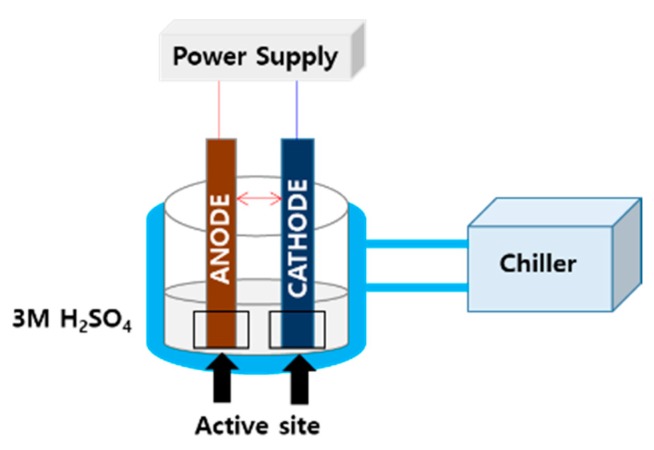
Schematic of the reactor.

The electrochemical behavior of the electrodes was tested on a ZIVE electrochemical workstation (WBCS3000M2) from Won-A Tech. (Seoul, Korea) using a two-electrode system [26,27]. The reactor system was a batch reactor, and there was a reactor volume of 2.0 L for each electrode, as shown in Figure 1. The electrode size was 2 cm × 2 cm (real electrolysis area = 4 cm^2^). To reduce the test time, the ALT was employed under a high current density of 5000, 10,000, and 15,000 mA/cm^2^ in 3 M of H_2_SO_4_ solution. Maintaining a constant temperature is an important operation parameter for accurate ALT; thus, a coolant (chiller) was used to maintain the temperature at 20 °C. Similarly, the prepared electrode was regarded as the anode, and the titanium oxide plate was considered as the cathode. The distance of the electrode was maintained at 0.5 cm between the anode and cathode. The voltage was adjusted to be 2, 4 and 6 V during electrolysis. When the electrode potential increased in the range of 20–40% of the initial values, corrosion occurred on the titanium surface.

## 3. Results and Discussion

### 3.1. Characterization of the Pd-Based Electrodes

**Figure 2:**
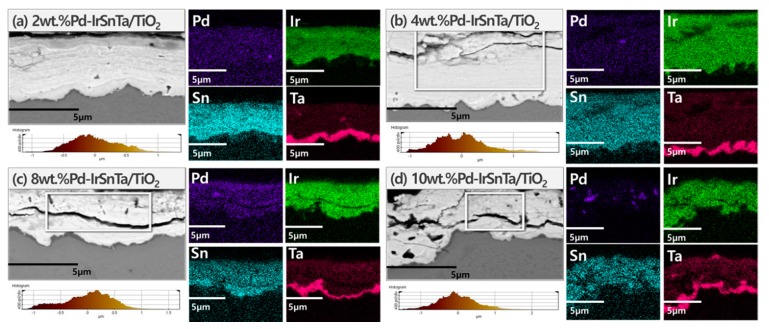
The Field Emission Scanning Electron Microscope (FE-SEM) image and surface roughness of electrodes.

The typical atomic and metal compositions obtained using a scanning electron microscope are listed in Table 1. Since the electrodes were manufactured by dip-coating, a slight error was discovered when the electrode was cut and analyzed for EDS in Figure 2.

The thickness of the substances coated on the electrode surface decreased as the amount of Pd increased. The SEM images illustrate that the microcracks and pores started to form as the amount of Pd deposition increased. In particular, cracks attributable to the pores were observed in the 10 wt.% Pd-IrSnTa/TiO_2_ electrode. The four components, PdO*_x_*, IrO*_x_*, SnO*_x_*, and TaO*_x_*, were evenly distributed, although TaO*_x_* was closely distributed on the surface of Ti_2_O_3_.

When the composition of the deposited electrodes’ cross section was compared to the theoretical target composition, approximately 10% of tantalum was planned to be deposited, and all four electrodes were deposited successfully. Pd was deposited at 2, 4, 8 and 10 wt.% with similar percentages of 1.3, 3.3 and 7.4 wt.%. However, the 10 g Pd-IrSnTa/TiO_2_ electrode has a large relative standard deviation (RSD) value. This indicates that Pd was excessively condensed on a certain part of the surface, and there is a section in the EDS image where the purple portion is clumped. The Ir and Sn were mixed with a 1:1 ratio; however, the ratio varied from 1.1–3.5 due to the interference effect of the four components. The surface of Pd-IrSnTa/TiO_2_ electrodes become rougher and less homogeneous as the Pd concentration increases.

### 3.2. Accelerated Life Testing

**Figure 3:**
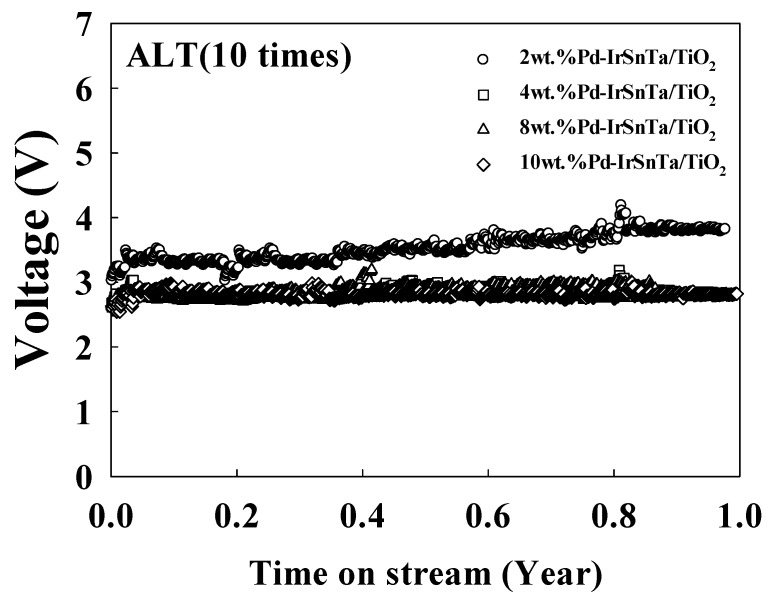
Variation of the electrode voltage with respect to time for the Pd-IrSnTa/TiO_2_ electrodes at 5000 mA/cm^2^.

The lifetime of the electrodes was tested using the manufactured catalyst. As shown in Figure 3, the lifetime of the electrodes for a duration of one year was tested with a current density of 5000 mA/cm^2^ and a 10 times acceleration rate.

The initial and final voltages at year one are listed in Table 2. This shows that the initial voltage decreased as the Pd increased. The voltage decreased in the order of 2 wt.% Pd-IrSnTa/TiO_2_, 4 wt.% Pd-IrSnTa/TiO_2_, and 8 wt.% Pd-IrSnTa/TiO_2_; however, the difference in the voltage is within the error range. When the lifetime of the electrodes for one year was tested at 5000 mA/cm^2^, the final voltage was similar to the initial voltage, excluding that for the 2 wt.% Pd-IrSnTa/TiO_2_ electrode. When the lifetime of the four electrodes for one year was tested at a 10 times acceleration rate, the final voltage was similar to the initial voltage regardless of the amount of Pd deposition. At a 10 times acceleration rate, the increase in voltage was 5%, 3%, 0%, and 0% as the amount of Pd deposition increased from 2 wt.% to 10 wt.%, respectively. In addition, the initial voltage was lower when the amount of Pd deposition increased.

Pd doping played an important role in the stability enhancement of the Pd-based electrode at the initial voltage values in our research. Li et al. mentioned that the presence of Pd enhanced the improvement of structure [29]. For the enhancement of the electrochemical oxidation ability of the electrode, there is the optimum ratio of Sn/Pd (about 2.5%).

**Figure 4:**
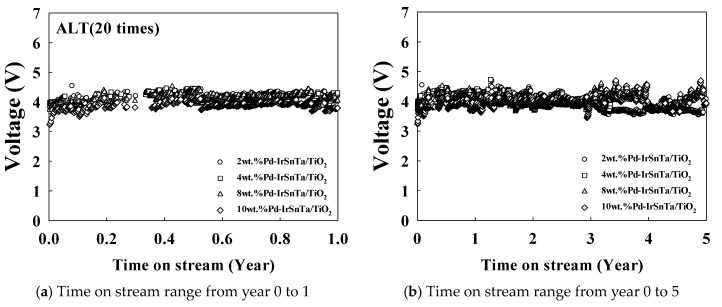
Variation of the electrode voltage over time for the Pd-IrSnTa/TiO_2_ electrodes at 10,000 mA/cm^2^, (**a**) Time on stream range from year 0 to 1; (**b**) Time on stream range from year 0 to 5.

Figure 4 illustrates the results of the same test being conducted at a 20 times acceleration rate with a current density of 10,000 mA/cm^2^. The results show that the initial voltage for the four electrodes increased as the current density increased. The 4 wt.% Pd-IrSnTa/TiO_2_ electrode had a relatively high initial voltage; however, other electrodes had the initial voltage within the error range. This result corresponds to the test result with a current density of 5000 mA/cm^2^.

Table 3 displays the results of the electrode lifetime test, with a current density of 10,000 mA/cm^2^. The results indicate that the voltage of the 2 wt.% Pd-IrSnTa/TiO_2_ and the 4 wt.% Pd-IrSnTa/TiO_2_ electrodes increased by 3.0% and 3.2%, respectively. Meanwhile, the 8 wt.% Pd-IrSnTa/TiO_2_ and the 10 wt.% Pd-IrSnTa/TiO_2_ electrodes increased by 5.5% and 9.3%, respectively. For the lifetime of the four electrodes over five years, the voltage did not increase by more than 40%. However, the increase in voltage after five years was 7.9%, 8.7%, 11.9%, and 10.0% higher than the initial voltage for 2, 4, 8 and 10 wt.%, respectively.

**Figure 5:**
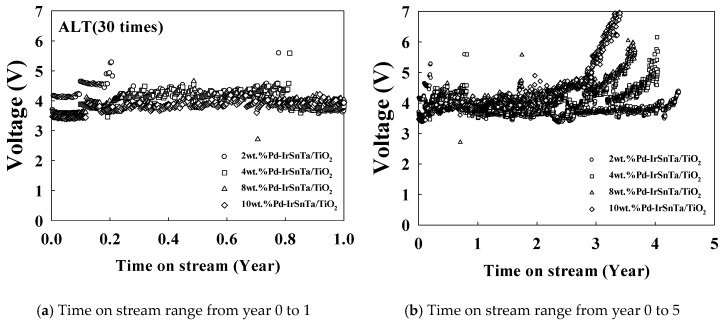
Variation of the electrode voltage with time for the Pd-IrSnTa/TiO_2_ electrodes at 15,000 mA/cm^2^ of current density; (**a**) Time on stream range from year 0 to 1; (**b**) Time on stream range from year 0 to 5.

Figure 5 depicts the results of the same test being conducted at a 30 times acceleration rate and a current density of 15,000 mA/cm^2^. The results show that the voltage vibration is greater than the test being conducted with an acceleration rate of 10 and 20 times. It can be inferred that the voltage easily increased as the amount of impurities extracted were significantly increased when the acceleration rate was further multiplied. This is attributed to the purple impurities that formed on the surface of the anode due to O_2_ bubbling during the electrowinning process. In previous studies, the purple impurities were determined to be Ir and Sn. Overall, the voltage increased with the multiplication of the acceleration rate.

Life testing for the electrodes was conducted at a current density of 15,000 mA/cm^2^ until the voltage increased by 40% or more, as shown in Table 4. The results demonstrate that the increase in voltage was mostly stable for the 2 wt.% Pd-IrSnTa/TiO_2_ electrode. In contrast, it was determined that the 4 wt.% Pd-IrSnTa/TiO_2_, the 8 wt.% Pd-IrSnTa/TiO_2_, and the 10 wt.% Pd-IrSnTa/TiO_2_ electrodes had a lifetime of 4.1, 3.7 and 3.5 years, respectively. This result demonstrates that the addition of Sn to the PdOx solution can improve the electrochemical stability [30].

The difference in the lifetime of the electrodes is due to the components of the electrodes. Ir is often used as the main component of the electrodes due to its long lifetime. Thus, considering the nature of the added components, the lifetime of Sn that contains Ir, which is known to have a considerable lifetime, will be longer.

The lifetime of the electrodes proceeded to shut down at a 30 times acceleration rate when the final voltage increased by 40% or more compared to the initial voltage. As a result, the difference was observed when the amount of Pd deposition varied. The voltage of the 10wt.% Pd-IrSnTa/TiO_2_ electrode increased first, and then, it decreased in the order of 8wt.% Pd-IrSnTa/TiO_2_, 4wt.% Pd-IrSnTa/TiO_2_, and 2wt.% Pd-IrSnTa/TiO_2_.

### 3.3. Pd-based Electrode after the Reactions

**Figure 6:**
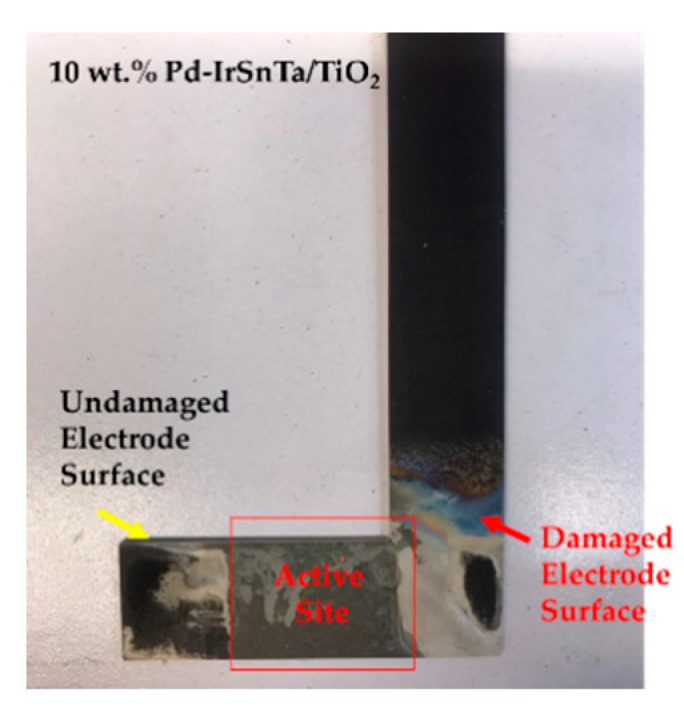
The 10 wt.% Pd-IrSnTa/TiO_2_ electrode after activation.

As shown in Figure 6, the electrodes used in the experiments were later photographed. As displayed in the pictures, severe corrosion occurred along the bottom of the anode. The parent metal titanium was not damaged at the bottom where the electrode color became lighter; however, the color of the membrane of the active electrode material also became lighter.

When the electrode surface components were analyzed after the ALT for the electrodes, the active electrode materials Pd, Ir, and Sn components decreased by about 70–80%. In contrast, the content of tantalum was almost the same [31,32]. In the SEM images, the surface cracks with holes were intermittently observed. The surface roughness (*R*q and *R*a) was increased with increasing palladium contents; however, the surface roughness (*R*q and *R*a) of Pd-IrSnTa/TiO_2_ electrodes are lower than before ALT. After ALT, the coating compositions on the surface were lost, and then the thickness and surface roughness were measured similarly.

It was not expected that the electrode loss would occur along the left and right sides, excluding the active surface of the electrodes, as they were supported by Teflon tape where electrolysis does not occur; however, the electrodes were lost [33].

**Figure 7:**
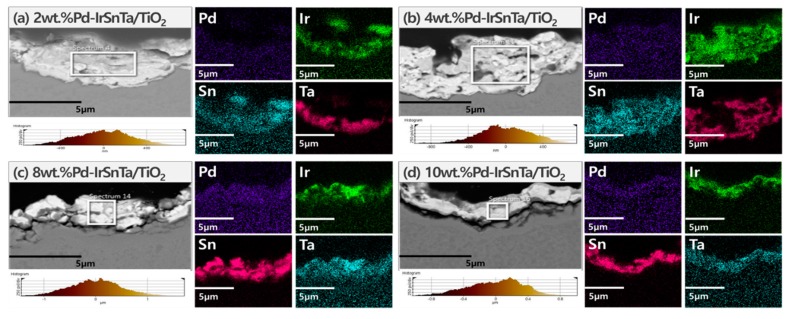
The SEM image and the surface roughness of Pd-IrSnTa/TiO_2_ electrodes after 30 times ALT.

Figure 7 shows the image of the electrode surface taken by the SEM. There is a boundary between the area where the electrode membrane is consumed, or desorption has failed to occur and the area where titanium is damaged after the electrode membrane has detached.

According to the surface composition analysis of the four electrodes that were tested at a 30 times acceleration, the Pd content decreased from 0.4% to 0.2%, as shown in Table 5. This indicates that about 90% of the Pd was detached from the electrode surface. Specifically, the ratio of Ir and Sn increased from two to four as the Pd content increased. The initial coating composition had a ratio of 3:1; however, the mixing ratio of Ir and Sn was abnormal, which may have caused the instability as the two components affected the stability, as seen in Table 6.

As the multiple of acceleration increased, the content of the four components on the electrode surface after activation for one year decreased. In particular, the content of Pd significantly dropped. Additionally, Ir and Sn also decreased along with Pd in the test conducted at a 30 times acceleration rate.

**Figure 8:**
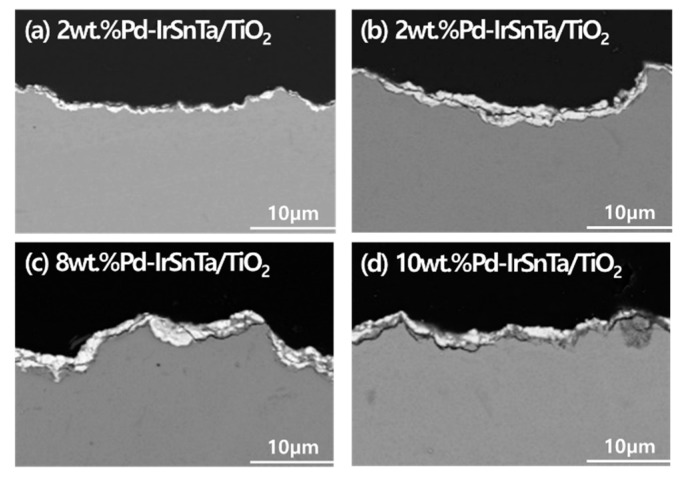
SEM image of Pd-IrSnTa/TiO_2_ electrode after activation for five years.

Since the acceleration rate has a major effect on the content of the electrode surface, an appropriate multiple of acceleration is required for the experiments. The thickness and form of the electrodes were inspected after life testing. Figure 8 show that almost all the electrodes had a 2 µm-thick coating substance remaining, regardless of the composition of the electrodes. Compared to the initial thickness, the 2 wt.% Pd-IrSnTa/TiO_2_ electrode had 78%, the 4 wt.% Pd-IrSnTa/TiO_2_ electrode had 70%, and both the 8 wt.% Pd-IrSnTa/TiO_2_ and 10 wt.% Pd-IrSnTa/TiO_2_ electrodes had 50% of their thickness etched. The substrate TiO_2_ and the four components were evidently detached for the 8 wt.% Pd-IrSnTa/TiO_2_ and 10 wt.% Pd-IrSnTa/TiO_2_ electrodes.

## 4. Conclusions

The accelerated life testing of the electrodes was conducted for electrodes of small sizes, thus reducing the load of a power supply device. In this study, the area of the electrode was set to 4 cm^2^ for the experiments. The voltage was set high with a low electrolyte concentration to elevate the current density in order to create the worst-case scenario. The electrolyte used in this study was 3 M H_2_SO_4_, and the current density was set to 5000, 10,000 and 15,000 mA/cm^2^ for the experiments.

The temperature of water was maintained at 20 °C using a cooler. When the current was constant, active electrode components were consumed and detached as the voltage applied to the electrodes increased. When the voltage increased by 2 V, the surface of the parent metal titanium also started to corrode. The loss of the electrode began from both ends instead of the center.

The results of the study infer that setting the multiple of acceleration too high can cause the inherent performance of the electrodes to abruptly degrade. This action makes it difficult to deduce the actual performance. Therefore, the multiple of acceleration must be set by considering the composition of the electrodes and other substances that can affect the lifetime of the electrodes after conducting basic screening tests. When the test was performed at a 10 times acceleration rate, the voltage value was stable; however, this takes more time to complete the test. For stability, a 20 times acceleration rate is appropriate. When the test was performed at a 30 times acceleration rate, the damage in the electrodes was the greatest, thus resulting in a shorter lifetime than the usual lifetime of the electrodes.

The amount of Pd affects the voltage. As the amount of Pd deposition increases, the initial voltage is lower. During life testing, as the amount of Pd increased, the content of Ir and Sn, which affects the lifetime, was reduced. This ultimately shortens the lifetime of the electrodes. Consequently, the 2 wt.% Pd-IrSnTa/TiO_2_ electrode has the longest lifetime.

## Figures and Tables

**Table 1:** The thickness and the composition of the Pd-based electrodes.

Electrode	Thickness (um)	Composition (%) ± R.S.D				Roughness	
		Pd	Ir	Sn	Ta	*R*q (µm)	*R*a (µm)
2wt.%Pd-IrSnTa/TiO_2_	10.06 ± 2.18	1.33 ± 0.39	34.64 ± 9.35	13.71 ± 0.12	10.08 ± 0.86	0.384	0.308
4wt.%Pd-IrSnTa/TiO_2_	7.69 ± 0.05	3.30 ± 1.50	30.49 ± 6.87	21.89 ± 3.25	9.72 ± 0.42	0.498	0.388
8wt.%Pd-IrSnTa/TiO_2_	4.9 ± 0.60	7.41 ± 1.41	34.54 ± 6.95	9.86 ± 3.99	10.22 ± 2.82	0.517	0.460
10wt.%Pd-IrSnTa/TiO_2_	4.3 ± 1.20	12.17 ± 3.74	33.43 ± 2.67	13.92 ± 2.26	11.86 ± 3.13	0.567	0.433

**Table 2:** Initial and final voltage of Pd-based electrodes at 5000 mA/cm^2^ for the current density.

	Initial Voltage	Final Voltage at Year 1	Resistance (ohm)
2 wt.%Pd-IrSnTa/TiO_2_	3.35	4.05	2.02
4 wt.%Pd-IrSnTa/TiO_2_	2.87	2.95	1.43
8 wt.%Pd-IrSnTa/TiO_2_	2.79	2.80	1.49
10 wt.%Pd-IrSnTa/TiO_2_	2.76	2.77	1.49

**Table 3:** Initial and final voltage of the Pd-based electrodes with a current density of 10,000 mA/cm^2^.

	Initial Voltage	Final Voltage at Year 1	Final Voltage at Year 5
2 wt.%Pd-IrSnTa/TiO_2_	3.84	3.96	4.17
4 wt.%Pd-IrSnTa/TiO_2_	3.88	4.01	4.25
8 wt.%Pd-IrSnTa/TiO_2_	3.79	4.01	4.30
10 wt.%Pd-IrSnTa/TiO_2_	3.62	3.99	4.02

**Table 4:** Initial and final voltage of Pd-based electrodes with a current density of 15,000 mA/cm^2^.

	Initial Voltage	Final Voltage at Year 1
2 wt.%Pd-IrSnTa/TiO_2_	4.17	4.30
4 wt.%Pd-IrSnTa/TiO_2_	3.61	3.73
8 wt.%Pd-IrSnTa/TiO_2_	3.52	3.92
10 wt.%Pd-IrSnTa/TiO_2_	3.46	3.94

**Table 5:** The thickness, composition, and roughness of Pd-IrSnTa/TiO_2_ electrodes after 30 times ALT.

Electrode	Thickness (µm)	Composition (%) ± R.S.D				Roughness	
		Pd	Ir	Sn	Ta	*R*q (µm)	*R*a (µm)
2wt.%Pd-IrSnTa/TiO_2_	1.8–2.6	0.41 ± 0.14	5.36 ± 0.63	4.99 ± 0.90	13.44 ± 3.17	0.256	0.207
4wt.%Pd-IrSnTa/TiO_2_	1.7–2.8	0.37 ± 0.05	6.31 ± 4.03	3.43 ± 1.58	12.19 ± 1.21	0.275	0.223
8wt.%Pd-IrSnTa/TiO_2_	1.7–2.9	0.42 ± 0.02	3.10 ± 0.34	1.73 ± 0.12	10.68 ± 0.69	0.304	0.248
10wt.%Pd-IrSnTa/TiO_2_	1.8–2.2	0.18 ± 0.01	3.92 ± 2.62	1.10 ± 0.40	8.63 ± 0.28	0.336	0.274

**Table 6:** The composition of the 8 wt.% Pd-IrSnTa/TiO_2_ electrode after activation for five years.

	Pd	Ir	Sn	Ta
Raw materials	7.41 ± 1.41	34.54 ± 6.95	9.86 ± 3.99	10.22 ± 2.82
20 times	0.60 ± 0.34	13.61 ± 4.72	4.67 ± 1.23	8.96 ± 1.16
30 times	0.18 ± 0.01	3.92 ± 2.62	1.10 ± 0.40	8.63 ± 0.28
